# Cross-Sectional Analysis of the Utility of Pulmonary Function Tests in Predicting Emphysema in Ever-Smokers

**DOI:** 10.3390/ijerph8051324

**Published:** 2011-04-29

**Authors:** Sean E. Hesselbacher, Robert Ross, Matthew B. Schabath, E. O’Brian Smith, Sarah Perusich, Nadia Barrow, Pamela Smithwick, Manoj J. Mammen, Harvey Coxson, Natasha Krowchuk, David B. Corry, Farrah Kheradmand

**Affiliations:** 1 Division of Pulmonary, Critical Care, and Sleep Medicine, Department of Medicine, Baylor College of Medicine, One Baylor Plaza, Houston, TX 77030, USA; E-Mails: hesselba@bcm.tmc.edu (S.E.H.); rross@bcm.edu (R.R.); perusich@bcm.edu (S.P.); nadiabarrow@gmail.com (N.B.); pamelas@bcm.edu (P.S.); mammen@buffalo.edu (M.J.M.); dcorry@bcm.edu (D.B.C.); 2 Division of Cancer Prevention and Control, The H. Lee Moffitt Cancer Center and Research Institute, 12902 Magnolia Drive, Tampa, FL 33612, USA; E-Mail: Matthew.Schabath@moffitt.org; 3 Children’s Nutrition Research Center (CNRC), Baylor College of Medicine, 1100 Bates St., Houston, TX 77030, USA; E-Mail: esmith@bcm.edu; 4 University of British Columbia, Vancouver Campus, 2329 West Mall, Vancouver, BC V6T 1Z4, Canada; E-Mails: Harvey.Coxson@vch.ca (H.C.); Natasha.Krowchuk@vch.ca (N.K.); 5 Michael E. DeBakey VA Medical Center, 2002 Holcombe Blvd, Houston, TX 77030, USA

**Keywords:** airflow limitation, chronic obstructive pulmonary disease, CT morphometry, emphysema, airway wall thickness, pulmonary function test

## Abstract

Emphysema is largely an under-diagnosed medical condition that can exist in smokers in the absence of airway obstruction. We aimed to determine the sensitivity and specificity of pulmonary function tests (PFTs) in assessing emphysema using quantitative CT scans as the reference standard. We enrolled 224 ever-smokers (current or former) over the age of 40. CT of thorax was used to quantify the low attenuation area (% emphysema), and to measure the standardized airway wall thickness. PFTs were used individually and in combination to predict their ability to discriminate radiographic emphysema. Significant emphysema (>7%) was detected in 122 (54%) subjects. Twenty six (21%) emphysema subjects had no evidence of airflow obstruction (FEV_1_/FVC ratio <70%), while all subjects with >23% emphysema showed airflow obstruction. The sensitivity and specificity of spirometry for detecting radiographic emphysema were 79% and 75%, respectively. Standardized airway wall thickness was increased in subjects with airflow obstruction, but did not correlate with emphysema severity. In this cohort of lifetime ever-smokers, PFTs alone were inadequate for diagnosing emphysema. Airway wall thickness quantified by CT morphometry was associated with airflow limitation, but not with emphysema indicating that the heterogeneous nature of lung disease in smokers may represent distinct phenotypes.

## 1. Introduction

The prevalence of cigarette smoking continues to rise in most developing countries around the world, but in the United States, former smokers now outnumber current smokers [1–3]. Despite smoking cessation, recent demographic data suggest that many ever-smokers (herein defined as former or current smokers) develop chronic obstructive pulmonary disease (COPD) [4,5]. A complete phenotypic characterization of lung disease in smokers is often delayed until the onset of self-reported clinical symptoms and findings of airflow obstruction on pulmonary function tests (PFTs), which further obscures the true prevalence and extent of tissue damage in the smoking population. While newer studies increasingly document the genetic and immune factors critical to the underlying pathogenesis of COPD, a comprehensive understanding of lung parenchymal destruction in ever-smokers remains elusive [6–8].

An exaggerated reduction in Forced Expiratory Volume in 1 second (FEV_1_), either absolute or expressed as a trend over time, has been shown to have an inverse association with life expectancy [9] and previous studies have indicated that airflow limitation correlated directly with qualitative measurements of emphysema in ever-smokers [10–12]. However the degree of airflow obstruction, if any, is highly variable even among ever-smokers with equal pack year histories [13].

The American Thoracic Society (ATS) and European Respiratory Society (ERS) have advocated the use of the lower limit of normal (LLN) based on the National Health and Nutrition Examination Survey (NHANES) data on lung function in a healthy population [14,15] to diagnose COPD. Alternatively, the Global Initiative for Chronic Obstructive Lung Disease (GOLD) has in the last decade promoted the use of a fixed FEV_1_/FVC ratio of less than 70% to diagnose COPD [16]. This latter guideline is now widely used to classify smokers with and without COPD. Based on lung function and symptoms, smokers are categorized into one of four GOLD stages: mild, moderate, severe and very severe [16]. There is a lack of knowledge of the prevalence and natural history of lung parenchymal destruction as seen in emphysema. The importance of determining the presence of emphysema in ever smokers, independent of airway obstruction, has recently become more recognized [17]. In particular, emphysema has been found to be an independent risk factor for cardiovascular diseases [18] and is an under-detected clinical condition in ever-smokers. Epidemiological studies have shown that lung cancer risk is strongly related to radiographic emphysema, independent of airflow obstruction [19–22]. Recently released data from the National Lung Screening Trial show that CT scan-based screening of over 53,000 smokers reduced lung cancer deaths by 20%; however obtaining CT scans may not be a feasible option for all smokers given the risk of radiation exposure and cost concerns [23,24]. Therefore, evaluation of currently available tests (such as PFT and spirometry) that may discriminate smokers with emphysema are much needed to better identify those who are at a higher risk for lung cancer, and who would benefit most from CT scan-based cancer surveillance.

Past studies have demonstrated that CT diagnosis by visual assessment is closely correlated with pathologic emphysema grade [25,26] and allows for accurate diagnosis of emphysema. Further, qualitative assessment of emphysema severity has been shown to relate to FEV_1_ impairment and airway wall thickness (AWT) [27]. AWT can also be quantified by CT imaging, and has recently been shown to correlate with symptoms in COPD patients [28,29]. To gain further insight into the relationships between airflow obstruction and emphysema, we designed a study to determine accuracy of pulmonary function testing in the diagnosis of emphysema, as measured by CT morphometry, in a cohort of ever-smokers [30,31]. A second aim of the study was designed to evaluate the relationship of airway wall thickness (AWT), with airflow obstruction and with the degree of (%) emphysema. A portion of this work was recently reported as an abstract [32].

## 2. Materials and Methods

### 2.1. Clinical and Demographic Characteristic of the Study Participants

We prospectively recruited 224 ever-smokers as part of the Longitudinal Exacerbation Study of COPD (LES-COPD). Enrollment criteria included age over 40, no history of concurrent lung cancer, chest surgery, or chronic lung diseases other than COPD (e.g., sarcoidosis, fibrosis, *etc*.). Random participants that met our inclusion/exclusion criteria were enrolled from local newspaper advertisements and at three clinics within the Texas Medical Center, in Houston, Texas: the Ben Taub General Hospital, Baylor Clinic, and Michael E. DeBakey Veterans Administration (VA) hospital. All studies were approved by the Institutional Review Board at Baylor College of Medicine (H-18029; Longitudinal Exacerbation Study of COPD) and written informed consent was obtained from all study participants.

### 2.2. Phenotypic Characterization of Former and Current Smokers

All pulmonary function tests were performed in diagnostic laboratories (Michael E. DeBakey VA and BCM clinics) that use standardized equipments and follow ATS/ERS guidelines; if the FEV_1_ was reduced below 80% of predicted or FEV_1_/FVC was below 70%, the participants received two doses of bronchodilator (albuterol 180 microgram) and a repeat measurement of spirometry was performed according to the ATS/ERS guidelines [14]. Calculation of all pulmonary function tests, in relation to reference values, were performed using computer programs and reviewed by trained physicians, without knowledge of the CT morphometry results. Normative values for spirometry were based on NHANES III data and absolute lung volumes were measured using plethysmograph [33]. Lung volume normative values were based on the equations endorsed by the ERS [15], with the upper limits of normal (ULN) calculated based on the 95% confidence interval. The current ATS/ERS guidelines [14], do not recommend a single reference equation for the DLCO, due to measurement variability among laboratories; we calculated our normal values based on three representative equations: Crapo [34], Cotes [35], and Miller [36], and used the latter equation as the most optimal predictive values with the lower limit of normal (LLN) calculated based on the 95% confidence interval.

### 2.3. Quantitative CT Morphometry Assessment of Emphysema

CT scans of the lung were acquired with the subject in supine position during end-inspiratory breath holding using a Siemens Cardiac Sensation Cardiac scanner (Siemens Medical Solutions). The subjects were coached to take a full breath, with imaging parameters of 120 kVp, 130 100 mAs and a pitch of 1.75. The images were reconstructed at 1 and 5 mm thickness using both an intermediate (b35f) and a high (b65f) spatial frequency reconstruction algorithm. The de-identified CT images were archived on to CDs, sent to the University of British Columbia (UBC), and were analyzed using the previously validated EmphylxJ custom software [30,37]. Briefly, emphysema was assessed by segmenting the lung parenchyma from the chest wall and large central blood vessels using a modified border tracing with a prior position-knowledge algorithm. A value of greater than 7% Low Attenuation Area (%LAA) below −950 HU was used as the upper limit of non-emphysematous lung; this value has been carefully validated with macroscopic and microscopic evaluation of lung tissue, which found that 6.8% LAA represents the upper limit of non-emphysematous lung [25,26,37,38].

### 2.4. Measurement of Airway Wall Thickness

The airway wall thickness (AWT) was determined using the algorithm as previously devised at iCAPTURE center at UBC [39]. Briefly, airways that were cut in an acceptable cross section (long/short internal diameter 2.2 or less) were identified and AWT analysis was performed using the 1 mm thick CT images. Every 10^th^ image was chosen using the spatial frequency reconstruction algorithm CT (b65f), to sample unique airways; on average 39 ± 23 airways per subject were measured (minimum = 5, maximum = 118). Briefly, a seed point was placed in the lumen of the airway, and radial lines were drawn to find the internal and external points at which the X-ray attenuation value was half the maximum value within the wall as we have described [40,41]. A standardized measurement of AWT was obtained to minimize sampling bias; the measurements of internal perimeter (Pi) was correlated with the square root of airway wall area for each subject as described [40,41].

### 2.5. Statistical Analysis

Since the data were not normally distributed, the degree of linear association between two continuous measures was assessed using Pearson’s correlation coefficient. Comparisons between unmatched pairs were performed using the Mann-Whitney U non-parametric test. Test characteristics for PFT (sensitivity, specificity, likelihood ratios, and predictive values) were calculated using previously described formulas [15,42] and using CT quantified emphysema (%LAA) with ≥7% as the reference standard [25,26,37,38]. Test characteristics were also calculated for combinations of PFTs, whereas if any component of the combination is positive, the combination is considered positive. Each combination includes FEV_1_/FVC < 70%, as this is the current standard for diagnosis of COPD. Positive (PPV) and negative (NPV) predictive values were also calculated based on this study population, as the exact prevalence of anatomic emphysema in ever-smokers has not yet been defined in the U.S. or worldwide populations. Statistical significance was defined as a p value less than 0.05.

## 3. Results

### 3.1. Demographics Distributed by GOLD Classification and CT Evidence of Emphysema

**Figure 1.**
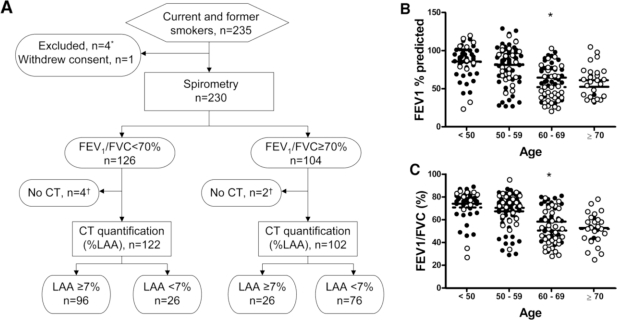
Schematic overview of the study design and FEV_1_% predicted and FEV_1_/FVC ratio among ever-smokers. (**A**) Flowchart of the recruitment, exclusions, dropouts, measurements, and results in evaluating the diagnostic accuracy of spirometry (FEV_1_/FVC < 70%) in the detection of emphysema, as defined by CT quantification (Low Attenuation Area; LAA ≥ 7%) is shown. The number of participants recruited, excluded, and measured is shown within the respective boxes. ^#^ Four subjects were excluded (three for concomitant lung disease; one for α-1-antitrypsin deficiency). ^¶^ Six total subjects did not have CT performed (two expired; three lost to follow-up; one withdrew consent). (**B**) FEV_1_% predicted was plotted by age group among current (N = 133, solid circles) and former (N = 91, open circles) smokers. (**C**) FEV_1_/FVC ratio was plotted by age group among current (N = 133, solid circles) and former (N = 91, closed circles) smokers. The group means are depicted by solid (current smokers) and dashed (former smokers) lines. * P < 0.05 current smokers *versus* former smokers.

In all, 235 ever smokers were enrolled, of which five were excluded or withdrew consent. Six subjects performed PFT, but failed to obtain CT scan, therefore the analyses included data collected from 224 subjects that completed both PFT and CT (Figure 1A). The clinical and demographic characteristics of study subjects, categorized by GOLD criteria, are shown in Table 1.

A total of 102 participants did not meet GOLD criteria for COPD (*i.e.*, FEV_1_/FVC < 70%) and were thus labeled as not GOLD classified (NGC). When FEV_1_% predicted and FEV_1_/FVC ratio (%) were plotted by age group [Figures 1(B,C)], we found that individuals >60 years of age had more severe disease than the younger individuals, though each group is well represented in our study population.

The demographic and clinical characteristics of the study subjects, categorized by presence or absence of CT-based emphysema using low attenuation area (LAA) are shown in Table 2. The stratification that distinguishes patients with significant emphysema defined as ≥7% in ever-smokers is based on published studies using a healthy cohort as control subjects where CT images were analyzed using custom software (EmphylxJ) as previously described [43,44]. Slightly older subjects and predominantly former smokers were over-represented in the emphysema group, compared with the group without emphysema. Those with emphysema ≥7% had significantly lower FEV_1_% of predicted, FEV_1_/FVC ratios and DLCO% of predicted than those without significant emphysema.

### 3.2. Diagnostic Value of Pulmonary Function Tests in Emphysema

**Figure 2.**
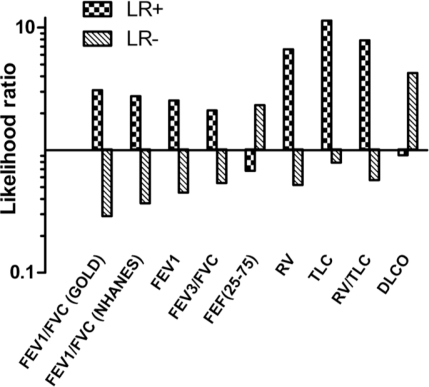
Likelihood ratios (LR) for detection of emphysema from pulmonary function tests. The positive (+) and negative (−) likelihood ratios for detection of emphysema as assessed by individual PFTs are shown where a LR+ of >10, or LR− of <0.1 represent conclusive increase in the likelihood of the presence, or absence of emphysema respectively.

We next measured the sensitivity and specificity of individual pulmonary function tests for predicting emphysema (LAA ≥ 7%) (Table 3). Positive and negative likelihood ratios (LR+, LR− respectively), and positive and negative predictive values (PPV and NPV respectively) for each pulmonary function test were also determined (Figure 2).

The sensitivity and specificity of FEV_1_/FVC < 70% were 79% and 75%, respectively for detecting emphysema. Similar sensitivity and specificity were found when using NHANES reference values (73% and 74% respectively), indicating that both cutoff values are similar for the detection of significant emphysema in our cohort. The PPV and NPV for FEV_1_/FVC < 70% were 79% and 75%, respectively. The only pulmonary function test that met the threshold (LR+ > 10; LR− < 0.1) for a conclusive test in identifying emphysema was, TLC > upper limit of normal (ULN) (Figure 2).

However in the absence of airflow obstruction, the TLC was abnormal only three times, yielding a specificity of 99% but a sensitivity of only 8%. Combining pulmonary function tests proved no better than individual tests in discriminating emphysema (Table 4). As expected, increasing the number of abnormal tests increased the sensitivity of discriminating emphysema but resulted in drastically reduced specificity. For example, the combination of airflow obstruction (FEV_1_/FVC < 70%), elevated lung volumes (RV > ULN and TLC > ULN), and reduced DLCO (<LLN) resulted in 95% sensitivity. However, the specificity for emphysema using this combination dropped to 21%. None of the combinations met likelihood ratio criteria for a definitive test and none of the combinations out-performed an FEV_1_/FVC < 70% with regards to LR+.

The predictive value of FEV_1_/FVC < 70% and TLC>ULN were only 21% sensitive, but was 99% specific for emphysema with LR+ of 21.07 and PPV of 96%. Combining FEV_1_/FVC < 70% with other lung volume measurements, such as RV > ULN (LR+ 16.86, PPV 95%) or RV/TLC > ULN (LR+ 15.45, PPV 95%) produced similar results. The combination of all spirometry measures (FEV_1_/FVC, FEV_1_, FEV_3_/FVC, and FEF_25–75_) produced a sensitivity of 48% and specificity of 88%, while combining FEV_1_/FVC < 70% and DLCO < LLN produced a sensitivity of 74% and specificity of 80%.

### 3.3. Correlation between CT Emphysema and Pulmonary Function Tests

**Figure 3.**
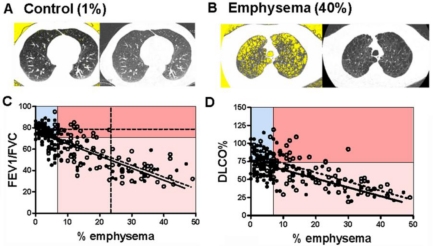
Correlation between PFTs and % emphysema in ever-smokers. Representative quantitative CT images of lung (left images) are matched to conventional CT images of the same lung (right images) from individuals without (**A**) and with (**B)** emphysema. Yellow background within lung margins (left images) was quantified as a percent of whole lung to determine the percent emphysema (1% and 40%, respectively; see Methods). (**C**) FEV_1_/FVC ratio was plotted against % emphysema, with regression lines in current (N = 133; solid line) and former (N = 91; dashed line) smokers. P < 0.0001; r = −0.7136 and r = −0.7574 Goodness of Fit for current and former smokers respectively. The lines are similar in slope (P = 0.78) and elevation (P = 0.17). The graph is divided into quadrants based on cutoff values for FEV_1_/FVC (70%) and % emphysema (7%). Additional dashed lines identify FEV_1_/FVC 78% (horizontal) and 23% emphysema (vertical). (**D**) DLCO% predicted plotted against % emphysema with regression lines in current (N =126; solid line) and former (N =89; dotted line) smokers. P < 0.0001; r = −0.4690 and r = −0.7074 Goodness of Fit for current and former smokers respectively. The lines are significantly different in elevation (p = 0.0003) but not slope (P = 0.79). The graph is separated into quadrants based on cutoffs for DLCO% (75%) and % emphysema (7%).

We found a significant negative correlation between the severity of emphysema, as quantified by CT morphometry (%LAA, representative samples are shown in [Figures 3(A,B)]) with FEV_1_/FVC (Figure 3C), and DLCO% predicted (Figure 3D). The negative correlation between % emphysema and FEV_1_/FVC was virtually identical in current and former smokers but there was a significant difference in the Y intercept of the correlation line in current when compared to former smokers.

The FEV_1_/FVC ratio demonstrated a stronger correlation with % emphysema in current (r = −0.71, P < 0.0001) and former smokers (r = −0.76, P < 0.0001) than either FEV_1_% or DLCO%. All subjects with LAA > 23% (vertical dashed line) also demonstrated FEV_1_/FVC < 70%, with 26 subjects with FEV_1_/FVC ≥ 70% demonstrating significant radiographic emphysema (LAA range 7–23%). The mean age of this group was 52 ± 7 indicating that emphysema in these ever-smokers is not due to lung parenchymal senescence reported in the aging population [45]. While no reasonable FEV_1_/FVC cutoff excluded emphysema, a cutoff of 78% excluded 94% of ever smokers with emphysema (horizontal dashed line).

### 3.4. Airway Wall Thickness Is Associated with Airway Obstruction but Not Emphysema

**Figure 4.**
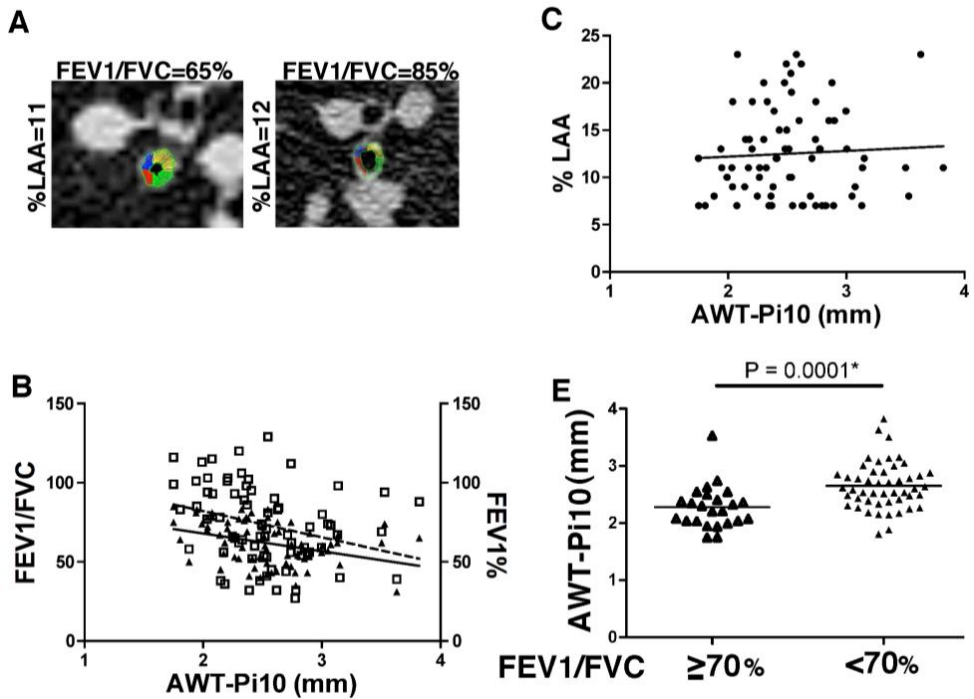
Airway wall thickness is increased in ever smokers with emphysema and airway obstruction. (**A**) A representative images of the internal perimeter of CT identified airway in subjects with similar degree of CT emphysema (11 and 12%) and with (left panel) or without (right panel) airflow obstruction. (**B**) Linear regression correlation of airway wall thickness (AWT-Pi10) was *versus* FEV_1_/FVC ratio (solid line; P = 0.0005; r = −0.3981) and FEV_1_% predicted (dashed line; P = 0.003; r = −0.3457) in 73 subjects with % emphysema between 7–23%. (**C**) AWT was plotted against % emphysema, with linear regression in the same group of 73 subjects. The correlation was not significant (P = 0.81, r = 0.0284). (**D**) Airway wall thickness (AWT-Pi10) measurements were assessed in 73 ever smokers with similar degree of emphysema (range 7 to 23%) comparing those with no airflow limitation (FEV_1_/FVC ≥ 70; N = 24) and with airflow obstruction (FEV_1_/FVC < 70; N = 49).

In this cohort, we determined that all ever-smokers with >23% emphysema had concurrent airflow obstruction. Therefore, we next analyzed the contribution of airway wall thickness (AWT-Pi10) to airflow obstruction and emphysema, in a subgroup of ever-smokers with >7% and < or equal to 23% emphysema (representative images shown in (Figure 4A).

This subgroup was similar to the overall study participants in terms of age (60 ± 11), pack-years (52 ± 36), FEV_1_/FVC (62 ± 13), FEV_1_ % predicted (73 ± 25). Analyses of AWT-Pi10 showed significant correlation between FEV_1_ % predicted (r = −0.40, P = 0.0005) and FEV_1_/FVC (r = −0.35, P = 0.0027), shown in Figure 4B, but not with %LAA (r = 0.03, P = 0.81, Figure 4C). When smoking status was taken into account, former smokers showed a significant correlation between AWT-Pi10 and FEV_1_ % predicted (r = −0.40, P = 0.009) and FEV_1_/FVC (r = −0.56, P = 0.0001), though these findings were not seen in current smokers (data not shown). Further, significantly higher AWT-Pi10 values were found in smokers with airflow obstruction (2.62 mm ± 0.42) compared to smokers without airflow obstruction (2.29 mm ± 0.40; P-value = 0.0001) (Figure 4D).

## 4. Discussion and Conclusions

In this study we determined the performance characteristics of standard PFTs in discriminating emphysema in a well-defined cohort of current and former smokers. We further evaluated the correlations between airway wall thickness, airflow obstruction, and emphysema in the same population. We found that airflow obstruction, as defined by an FEV_1_/FVC < 70%, was 79% sensitive and 75% specific in discriminating significant emphysema. Combining FEV_1_/FVC < 70% with FEV_1_ < LLN increased the sensitivity of emphysema detection to 80% but reduced specificity to 63%, while other combinations of PFTs decreased specificity further, and failed to improve predictive values. Measurement of lung volumes, specifically TLC, was the only method found to meet criteria for a definitive test, *i.e.*, a test in which a positive result would rule-in the presence of emphysema. However, an abnormally high TLC, in the absence of airflow obstruction, occurred rarely in our population, and therefore does not appear to have a practical application in clinical setting for detecting emphysema. Conversely, a reduced diffusion capacity was found to be quite sensitive for emphysema, however, this test is very non-specific as evidenced by a low LR+ < 1. Further current smokers had significantly reduced DLCO when compared to former smokers with the same degree of emphysema. This finding is consistent with the previously tested hypothesis that active smoking acutely reduces DLCO independent of emphysema by decreasing subjects’ lung capillary blood volume [46].

We show here that measurement of the FEV_1_/FVC ratio from simple spirometry demonstrated the best combination of sensitivity and specificity for discriminating emphysema and spirometry could be performed readily in clinics [15]. In a subset of subjects with less severe emphysema (LAA 7–23%), increased airway thickness was associated with airflow obstruction, suggesting that an increase in airway wall thickness significantly contributed to airflow obstruction. Further, the failure of emphysema severity to correlate with airway wall thickness in this subset suggests that airway disease and anatomical emphysema from smoking, at least in part, may represent distinct pathophysiological processes.

Our findings are of clinical importance to the study of COPD for many reasons. Currently, the exact prevalence of emphysema in smokers is unknown and we show here that, consistent with prior studies, emphysema remains an under diagnosed medical condition [47,48]. Further spirometry based classification of ever-smokers with smoking-related lung disease may fail to include those with emphysema who do not show reduced FEV_1_. Our findings here, together with those of others [48,49], highlight the importance of additional diagnostic tests that could provide an accurate estimate of concurrent emphysema in ever smokers. We show here that while standard pulmonary function tests correlate well with airway wall thickening, they fail to identify a substantial number of ever smokers with emphysema. These findings have important implications for the design of future clinical studies that should consider a better phenotypic classification of ever-smokers with radiographic assessment of emphysema.

Individuals with distinct patterns of anatomical emphysema may present with different degrees of pulmonary function abnormalities [50]. In this study we found no correlation between airway wall thickness and severity of emphysema, with the caveat that we did not measure the transpulmonary pressure to control for the elastic recoil pressure. Our findings thus represent an *in vivo* demonstration of the dissociation between airflow limitation, airway wall thickness and anatomical emphysema in smokers and suggest that the pathological processes underlying airway obstruction and emphysema are likely distinct. With a growing number of interventions and treatments targeted to patients specifically with emphysema [51,52], and our current findings that more advanced emphysema (LAA > 23%) is always associated with airflow obstruction, it is of increasing clinical importance that these patients are identified properly at an earlier stage of their disease.

Our findings underscore the need to develop improved diagnostic and prognostic methods for evaluating ever smokers who will eventually develop irreversible lung parenchymal disease. Chest CT evaluation is clearly the standard for detecting early emphysema *in vivo*, but is limited by cost and the long-term consequences of exposure to ionizing radiation [53]. Improved diagnostic methods are essential for answering critical questions regarding the natural history of smoking-related lung disease, specifically regarding the relationship of obstructive to destructive lung diseases and whether these processes represent inexorably progressive or self-limited conditions. A proposed strategy in the evaluation of ever smokers may be to measure full pulmonary function tests, including spirometry and lung volumes, and diffusion capacity; if spirometry is normal but hyperinflation, air trapping, or reduced gas exchange are noted, a CT should be considered to evaluate for isolated emphysema. Detection of emphysema without airflow obstruction may also provide an explanation of symptoms such as dyspnea or exercise intolerance in ever smokers with normal spirometry, who might otherwise be identified as normal. Future studies are necessary to identify emphysema at an early-stage, and smokers who are at risk of developing lung disease where intervention may be more efficacious. In summary, our data supports the notion that emphysema is a pathologic process that occurs in a substantial proportion of smokers independent of airflow obstruction, and thus highlighting the need for a modern approach to the evaluation of this large population of patients.

## Figures and Tables

**Table 1.** The clinical and demographic characteristics of study subjects, categorized by GOLD criteria.

**Stage**	**NGC (previously Stage 0)**	**COPD (GOLD)**			
		**(I)**	**(II)**	**(III)**	**(IV)**
Number	102	18	55	39	10
Age, mean (±s.d.)	53 ± 8	55 ± 9	64 ± 9	64 ± 9	59 ± 7
Height, cm (±s.d.)	170 ± 9	174 ± 11	174 ± 9	175 ± 8	173 ± 8
Weight, kg (±s.d.)	88 ± 21	79 ± 12	88 ± 18	80 ± 19	74 ± 16
Male, no. (%)	42 (41%)	14 (78%)	46 (84%)	35 (90%)	8 (80%)
Ethnicity, no. (%)					
Black	60 (59%)	7 (39%)	12 (22%)	13 (33%)	2 (20%)
Hispanic	4 (4%)	1 (6%)	1 (2%)	1 (3%)	1 (10%)
White	35 (34%)	13 (56%)	42 (76%)	25 (64%)	7 (70%)
Other	3 (3%)	0 (0%)	0 (0%)	0 (0%)	0 (0%)
Smoking status, no. (%)					
Current	72 (71%)	14 (78%)	29 (53%)	14 (36%)	4 (40%)
Former	30 (29%)	4 (22%)	26 (47%)	25 (64%)	6 (60%)
Pack-years, mean (±s.d.)	33 ± 30	40 ± 18	69 ± 41	66 ± 35	62 ± 24
Quitting years, mean (±s.d.)	14 ± 11	21 ± 11	12 ± 11	9 ± 10	9 ± 6
Lung Function					
% FEV_1_ (±s.d.)	92 ± 15	90 ± 7	64 ± 8	39 ± 5	25 ± 3
FEV1/FVC ratio (±s.d.)	78 ± 5	66 ± 4	56 ± 8	42 ± 8	34 ± 5
% DLCO (±s.d.)	78 ± 16	63 ± 20	59 ± 14	42 ± 15	35 ± 13

NGC: “Not GOLD Classified,” current and former smokers without airflow obstruction; s.d.: standard deviation; cm: centimeters; kg: kilograms; no.: number; %FEV_1_: forced expiratory volume in 1 second, percent of predicted (post-bronchodilator); FVC: forced vital capacity; %DLCO: diffusing capacity for carbon monoxide, percent of predicted; pack-years: number of years smoked multiplied by packs per day; quitting years: number of years since last reported smoking activity.

**Table 2.** Clinical and demographic information of the study participants when separated by CT quantification.

**CT Quantification**	**LAA < 7% No emphysema**	**LAA ≥ 7% Emphysema**
Number	102	122
Age, mean (±s.d.)	53 ± 8	62 ± 10
Height, cm (±s.d.)	170 ± 10	174 ± 9
Weight, kg (±s.d.)	85 ± 20	86 ± 19
Male, no. (%)	45 (44%)	100 (82%)
Ethnicity, no. (%)		
Black	59 (58%)	35 (29%)
Hispanic	4 (4%)	4 (3%)
White	37 (36%)	82 (67%)
Other	2 (2%)	1 (1%)
Smoking status, no. (%)		
Current	84 (82%)	49 (40%)
Former	18 (18%)	73 (60%)
Pack-years, mean (±s.d.)	41 ± 36	56 ± 36
Quitting years, mean (±s.d.)	13 ± 13	12 ± 10
Lung function		
%FEV1 (±s.d.)	87 ± 18	62 ± 26
FEV1/FVC (±s.d.)	74 ± 10	55 ± 16
%DLCO (±s.d.)	74 ± 16	57 ± 22

CT: computerized tomography; LAA: low attenuation area (% emphysema) by CT quantification; s.d.: standard deviation; cm: centimeters; kg: kilograms; no.: number; %FEV_1_: forced expiratory volume in 1 second, percent of predicted (post-bronchodilator); %DLCO: diffusing capacity for carbon monoxide, percent of predicted; pack-years: years smoked x packs per day; quitting years: number of years since last reported smoking activity.

**Table 3.** Individual pulmonary function tests as predictors of radiographic emphysema.

**Criteria**	**Sensitivity % (95% CI)**	**Specificity % (95% CI)**	**LR+**	**LR−**	**PPV %**	**NPV %**
FEV_1_/FVC < 70%	79 (71–85)	75 (65–82)	3.09	0.29	79	75
FEV_1_/FVC < LLN (NHANES)	73 (65–80)	74 (64–81)	2.76	0.37	77	68
FEV_1_< LLN	67 (59–75)	74 (64–81)	2.54	0.45	75	65
FEV3/FVC < LLN	62 (53–70)	71 (62–79)	2.11	0.54	71	61
FEF25–75 < LLN	68 (59–76)	43 (34–53)	0.68	2.32	59	54
RV > ULN	52 (43–61)	92 (86–96)	6.64	0.52	89	62
TLC > ULN	22 (16–30)	98 (94–100)	11.38	0.79	93	52
RV/TLC > ULN	46 (38–55)	94 (88–98)	7.87	0.57	90	60
DLCO < LLN	91 (84–95)	23 (16–33)	0.91	4.26	59	68

LLN: lower limit of normal; ULN: upper limit of normal; CI: confidence interval; LR+: positive likelihood ratio; LR−: negative likelihood ratio; PPV: positive predictive value; NPV: negative predictive value; %FEV_1_: forced expiratory volume in 1 second, % of predicted (post-bronchodilator); FVC: forced vital capacity; FEV_3_: forced expiratory volume in 3 seconds; FEF_25–75_: forced expiratory flow between 25% and 75% of expired FVC; RV: residual volume; TLC: total lung capacity, % of predicted; %DLCO: diffusing capacity for carbon monoxide, % of predicted.

**Table 4.** Combined pulmonary function tests as predictors of radiographic emphysema.

**Criteria**	**Sensitivity % (95% CI)**	**Specificity % (95% CI)**	**LR+**	**LR−**
FEV_1_/FVC < 70% and FEV_1_/FVC < LLN (NHANES)	79 (71–85)	74 (64–81)	2.97	0.29
FEV_1_/FVC < 70% and RV/TLC > ULN	79 (72–87)	72 (62–80)	2.79	0.29
FEV_1_/FVC <70% and FEV_1_ < LLN	80 (73–87)	63 (53–72)	2.16	0.31
FEV_1_/FVC < 70% and FEV_3_/FVC < LLN	80 (72–87)	65 (54–73)	2.27	0.31
FEV_1_/FVC < 70% and TLC > ULN	80 (72–87)	74 (64–81)	3.03	0.27
FEV_1_/FVC < 70% and RV > ULN	81 (73–87)	70 (60–78)	2.66	0.27
FEV_1_/FVC < 70% and RV > ULN and TLC > ULN	82 (74–88)	70 (60–78)	2.69	0.26
FEV_1_/FVC < 70% and FEF_25–75_ < LLN	84 (77–90)	38 (29–48)	1.36	0.41
FEV_1_/FVC < 70% and FEV_1_ < LLN and FEV_3_/FVC < LLN and FEF_25–75_ < LLN	87 (80–92)	27 (19–37)	1.20	0.48
FEV_1_/FVC < 70% and FEV_3_/FVC < LLN and DLCO < LLN	93 (87–97)	16 (10–25)	1.11	0.42
FEV_1_/FVC<70% and DLCO<LLN	93 (87–97)	21 (14–30)	1.19	0.32
FEV_1_/FVC < 70% and DLCO < LLN and RV/TLC > ULN	93 (87–97)	21 (14–30)	1.19	0.32
FEV_1_/FVC < 70% and DLCO < LLN and RV > ULN	94 (89–97)	21 (14–30)	1.20	0.28
FEV_1_/FVC < 70% and FEF_25–75_ < LLN and DLCO < LLN	95 (90–98)	13 (8–21)	1.09	0.39
FEV_1_/FVC < 70% and DLCO < LLN and TLC > ULN	95 (90–98)	21 (14–30)	1.21	0.24
FEV_1_/FVC < 70% and DLCO < LLN and RV > ULN and TLC > ULN	95 (90–98)	21 (14–30)	1.21	0.24
FEV_1_/FVC < 70% and FEF_25–75_ < LLN and DLCO < LLN and RV > ULN and TLC > ULN	97 (92–99)	13 (8–21)	1.11	0.26

* LR+ = positive likelihood ratio; LR− = negative likelihood ratio; LLN = lower limit of normal; ULN = upper limit of normal; FEV_1_ = forced expiratory volume in 1 second (post-bronchodilator); FVC = forced vital capacity; FEV_3_ = forced expiratory volume in 3 seconds; FEF_25–75_ = forced expiratory flow between 25% and 75% of expired FVC; RV = residual volume; TLC = total lung capacity; DLCO = diffusing capacity for carbon monoxide.
